# Beyond macrocirculation: comment on goal-directed hemodynamic therapy

**DOI:** 10.1590/1806-9282.20261121

**Published:** 2026-11-09

**Authors:** Faruk Yildiz, Volkan İnal

**Affiliations:** 1 Trakya University, Faculty of Medicine, Department of Internal Medicine, Division of Intensive Care Medicine – Edirne, Turkey.

Dear Editor,

We read with great interest the systematic review and meta-analysis by Silvinato et al., which pooled 35 randomized controlled trials and 4,903 patients to compare uncalibrated pulse contour analysis (UC-PCA)-guided hemodynamic therapy with standard of care in major or high-risk surgery^1^. Restricting inclusion to a single, well-defined monitoring technology is a genuine methodological strength. We propose that the null findings reflect the limits of macrocirculatory targets rather than a failure of the monitoring technology, and that two analytical choices make that null appear firmer than the data warrant.

Binary outcomes were pooled as Mantel-Haenszel risk differences (RDs)^1^. RD is baseline-risk dependent, and empirical work across 551 systematic reviews has shown that risk ratio and odds ratio models are on average more consistent across trials than RD when control-group event rates vary^2^. Current guidance accordingly favors relative measures for pooling, with absolute measures derived for presentation^3^; a large contemporary synthesis in this field reported all binary outcomes as odds ratios^4^. Across 35 trials in heterogeneous surgical populations, the reported RD for 90-day mortality (−0.00; 95%CI −0.02 to 0.01) may therefore reflect the summary statistic as much as the underlying biology. Relatedly, GRADE's updated imprecision guidance asks that the confidence interval of the absolute effect be judged against pre-specified decision thresholds^5^. No such threshold was defined, and the interval does not exclude a 1% absolute mortality difference (an effect most clinicians would consider important in major surgery). "A clinically important effect cannot be excluded" would be more defensible than "no difference," even at moderate certainty.

Intensive care unit length of stay was pooled despite I^2^=95% and rated very low certainty, yet the summary estimate (-0.44 days) is carried into the Evidence Synthesis and Conclusion^1^. At that level of inconsistency a common effect is difficult to defend, particularly because "goal-directed hemodynamic therapy" encompasses substantially different target variables and thresholds^4^. Moreover, 0.44 days and 1.24 h of ventilation fall below any plausible minimally important difference; statistical and clinical significance warrant separation in the conclusions. As the length-of-stay analyses relied on median-to-mean conversions that assume symmetry^6^ while length of stay is markedly right-skewed, a sensitivity analysis excluding converted trials would strengthen these estimates. We would also welcome the unrounded values for acute heart failure: the reported RD (−0.02; 95%CI −0.03 to −0.00) and number needed to treat (NNT) (40; 95%CI 24 to 125) are not reconcilable, since an NNT of 24 implies an absolute difference of 4.2%.

Our principal comment is physiological. UC-PCA optimizes macrocirculatory surrogates (stroke volume, stroke volume variation, cardiac index). Where hemodynamic coherence is lost, as it commonly is under inflammation and surgical stress, normalizing systemic variables does not translate into improved microcirculatory perfusion or tissue oxygenation^7^. This framework accounts for precisely the pattern the authors observed: benefit confined to fluid-balance-sensitive outcomes, and none in mortality, myocardial infarction, or acute kidney injury. Clinical data point the same way. Among 1,338 acute high-risk surgical patients, a low intraoperative peripheral perfusion index (PPI) was independently associated with severe postoperative complications or 30-day mortality, whereas a mean arterial pressure ≤65 mmHg was not (odds ratio 1.21; 95%CI 0.92–1.58; p=0.2)^8^. PPI is derived from the photoplethysmographic signal at no additional cost or invasiveness and reflects the interaction of vascular tone and stroke volume^9^.

The contrast with the resuscitation literature is now hard to ignore. In ANDROMEDA-SHOCK-2 (1,467 patients across 86 centers in 19 countries) a personalized resuscitation protocol targeting capillary refill time was superior to usual care for a hierarchical composite of mortality, duration of vital support, and hospital length of stay, driven mainly by shorter duration of vital support^10^. Read alongside the present review, the emerging signal is that what we aim at may matter more than what we measure it with. The authors’ null result is arguably not a failure of UC-PCA; it is evidence about the limits of macrocirculatory targets.

We therefore suggest that the next generation of perioperative goal-directed trials be defined not by device class but by target variable, and that they embed perfusion-based endpoints (PPI, capillary refill time, and lactate clearance) as co-primary targets alongside flow optimization, tested in risk-enriched populations. Framed this way, the authors’ careful synthesis is not a negative result but a useful boundary condition. We thank them for a rigorous and timely contribution.

## Data Availability

No datasets were generated or analyzed for this Letter to the Editor; all data discussed are available in the published articles cited in the reference list.
